# Rescue-like Behaviour in Mice is Mediated by Their Interest in the Restraint Tool

**DOI:** 10.1038/s41598-019-46128-5

**Published:** 2019-08-02

**Authors:** Hiroshi Ueno, Shunsuke Suemitsu, Shinji Murakami, Naoya Kitamura, Kenta Wani, Yu Takahashi, Yosuke Matsumoto, Motoi Okamoto, Takeshi Ishihara

**Affiliations:** 1 Department of Medical Technology, Kawasaki University of Medical Welfare, Okayama, 701-0193 Japan; 2 Department of Psychiatry, Kawasaki Medical School, Kurashiki, 701-0192 Japan; 3 Department of Neuropsychiatry, Graduate School of Medicine, Dentistry and Pharmaceutical Sciences, Okayama University, Okayama, 700-8558 Japan; 4 Department of Medical Technology, Graduate School of Health Sciences, Okayama University, Okayama, 700-8558 Japan

**Keywords:** Animal behaviour, Psychosis, Motivation

## Abstract

Acting without the expectation of compensation is called prosocial behaviour. Since prosocial behaviour requires high cognitive and social abilities, it has been thought to be only shown by primates. Although prosocial behaviour has been recently reported in rats, there are still questions regarding this finding. We demonstrated rescue-like behaviour in mice in a previous report. In this study, we investigated the motives underlying rescue-like behaviour for constrained cage-mates among mice. We prepared either a tube containing a ball of yarn or an opaque tube and assessed whether mice displayed the same rescue-like behaviour shown in the case of tube-restrained cage-mates. Mice did not open the lid of the tube containing the ball of yarn but opened the opaque tube lid. Mice showed a high interest in the tube in which the cage-mate had been restrained and prioritized staying in this tube rather than rescuing additional cage-mates. Oxytocin, which increases empathy, had no effect on the lid-opening behaviour. Thus, the rescue-like behaviour of mice is not based on empathy but is related to social interest in the cage-mate and the tube itself. These results suggest that rodent lid-opening behaviour may not conclusively prove the presence of prosocial behaviour.

## Introduction

Expressing socially desirable behaviours for conspecifics without external compensation is called prosocial behaviour^1^. Prosocial behaviour includes assistance behaviours, dispensing behaviours, and service activities. Prosocial behaviour is thought to be limited to primates showing signs of intelligence^2–4^, and the incidence of such behaviour in animals other than primates has not been elucidated.

Interestingly, rats, which are rodents, have been recently reported to exhibit prosocial behaviour. Ben-Ami *et al*. reported that rats could open the device lid when a cage-mate was confined in a transparent device^5^. Sato *et al*. demonstrated that rats attempt to open the door of a room for cage-mates soaked in water^6^. These reports suggest that rats display prosocial behaviour, and the published tests are now used to investigate prosocial behaviour in rodents^7^. Such rescue-like behaviour among rats is suggested to be based on empathy^5,6,8^, which is the ability to understand the feelings of a conspecific and share those feelings. Empathy is important for social animals^9^. The ability to recognize the state of a conspecific and act in its interest is profitable for the group and improves the chances of survival^10^. Empathy which is suggested as the basis of social behaviour has been historically considered to be a high-level cognitive process restricted to humans and primates. However, empathy has recently also been reported in non-primates^11–13^, birds^14,15^, and rodents^16–18^.

The lack of empathy is a feature of neuropsychiatric disorders such as autism, schizophrenia, and psychopathy^19–21^. The prosocial behaviour in rats is expected to be a very useful tool for elucidating the causes of these neuropsychiatric disorders and establishing therapeutic methods.

However, the rescue-like behaviour shown by rats in previous reports may not represent prosocial behaviour. The action of opening the lid of a cylinder constraining the conspecific may be motivated by the need for social contact with the cage-mate in the cylinder^22,23^. Similarly, opening the door for rats soaked in water may be motivated by an attempt to approach the water or by the rat’s interest in the cage-mate’s unusual behaviour^24^. Thus, a careful discussion is necessary before the conclusion can be drawn that rescue-like behaviour in rats represents empathy or prosocial behaviour^25^.

Previously, we reported for the first time that mice show rescue-like behaviours to help cage-mates restrained in tubes^26^. In our previous assessments, mice displayed rescue-like behaviours towards fellow mice that were not suffering. In addition, mice showed comparable interest in both restrained and free cage-mates. These results suggest that the rescue-like behaviour shown by mice may not be based on empathy unlike the rescue-like behaviour observed in rats. However, the motive for rescue-like behaviour in mice is not as clear as that in rats.

In this study, we investigated the motivations underlying the rescue-like behaviour shown by mice in the previous report. We analysed the behavioural changes in mice by modifying the experimental environment to determine whether this behaviour was motivated by empathy, social contact with the cage-mate, or interest in the restraining tube.

We also administered oxytocin to mice and examined the changes in rescue-like behaviour because oxytocin can enhance empathy in mice^27^. If the rescue-like behaviour was based on empathy, administration of oxytocin could be expected to influence the rescue-like behaviour.

## Results

### Effect of oxytocin on rescue-like behaviour

**Figure 1:**
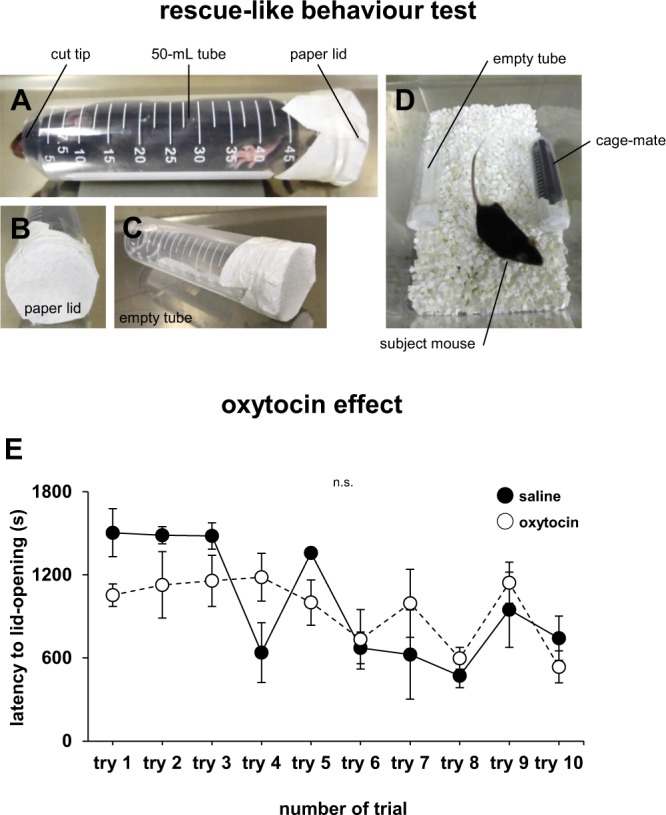
Rescue-like behaviour test and the effect of oxytocin on rescue-like behaviour. (**A**) Sample picture of a mouse waiting for its release from a 50-mL tube. (**B**) A sample image showing the back of a mouse-containing tube covered with a paper lid. (**C**) A sample image showing the back of an empty 50-mL tube covered with a paper lid. (**D**) Sample picture of the rescue-like behaviour test in the new home cage. (**E**) Rescue-like behaviour test for the constrained cage-mate: latency to paper lid-opening in each trial. Data are presented as the mean ± SEM. Statistical significance is represented by asterisks: *p < 0.05. n.s., not significant. The p values were calculated by two-way repeated measures ANOVA. n = 20 animals per test.

Oxytocin administration has been shown to have prosocial, anxiolytic, and anorexic actions. We investigated whether oxytocin affects rescue-like behaviour in mice. Mice underwent a rescue-like behaviour test 30 min after intraperitoneal injection of either saline or oxytocin. Constrained cage-mate mice were placed on one side of a new home cage with an empty tube being placed on the other side. The latency to lid-opening of the tube containing the cage-mate mouse was measured. We performed the test once a day for 10 days. Mice from both groups released the constrained mouse by gnawing on the paper lid. The latencies to lid-opening significantly decreased over the test period in both groups (Fig. 1E, oxytocin × trial: *F*_1,180_ = 1.384, p = 0.209; trials in control: *F* = 5.593, p < 0.001; trials in oxytocin: *F* = 1.921, p = 0.066). No significant intergroup difference was detected in the latency to lid-opening during the test period (Fig. 1E). Oxytocin did not affect rescue-like behaviour during the test period in these mice.

### Rescue-like behaviour depends on the tube condition

**Figure 2:**
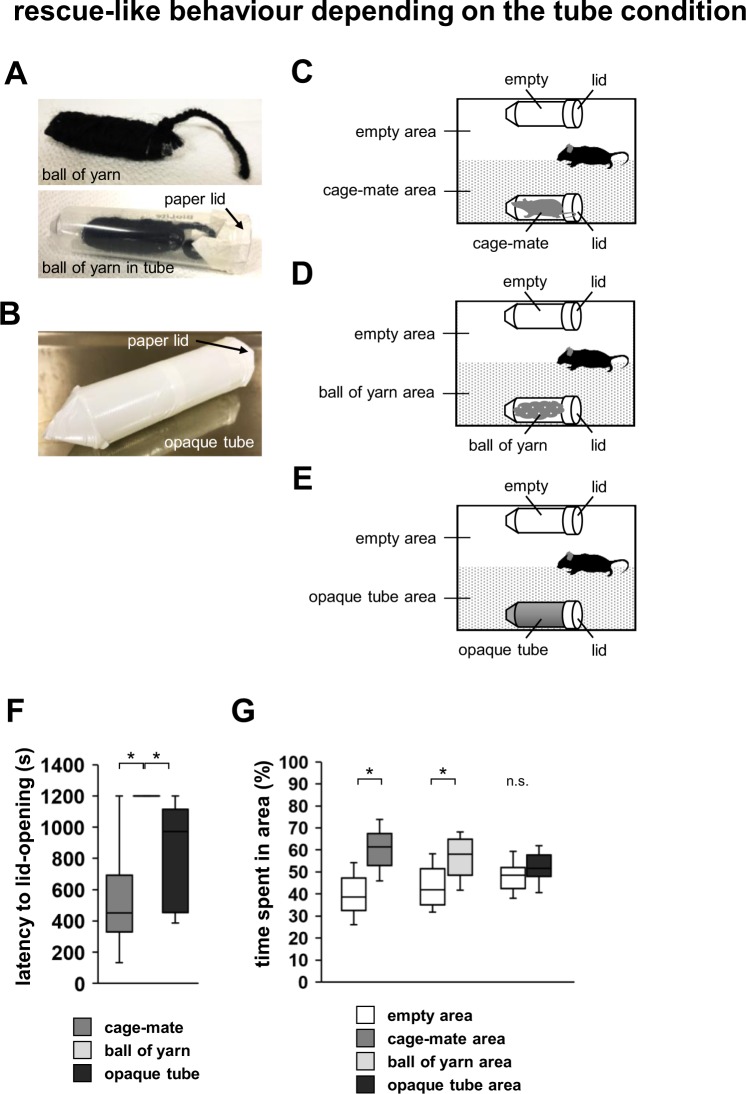
Rescue-like behaviour test depending on the tube condition. (**A**) Upper row: a sample image of the ball of yarn. Lower row: a sample image showing the back of the tube containing the ball of yarn with a paper lid. (**B**) A sample image of an opaque 50-mL tube with a paper lid. (**C**) Schematic diagram of the test. Rescue-like behaviour test for cage-mates: time spent in the cage-mate area or the empty area. (**D**) Rescue-like behaviour test for a ball of yarn: time spent in the ball of yarn area or the empty area. (**E**) Rescue-like behaviour test for the opaque tube: time spent in the opaque tube area or the empty area. (**F**) Comparison of the latency to lid-opening in the three tests. (**G**) Comparison of the time spent in each area of the three tests. All data are presented as box plots. Statistical significance is represented as *p < 0.05. n.s., not significant. The p values were calculated by one-way repeated measures ANOVA (**F**) and paired *t*-test (**G**). n = 10 animals per test.

We examined if the subject mice would show rescue-like behaviour when the tube contained a ball of yarn instead of a cage-mate mouse (Fig. 2B). We observed that the subject mice did not open the paper lid of the tube containing the ball of yarn (Fig. 2F, Supplementary Video S1, *F*_1,9_ = 282.938, p < 0.001; cage-mate vs. ball of yarn: p = 0.001; cage-mate vs. opaque tube: p = 0.503; ball of yarn vs. opaque tube: p = 0.024). The subject mice spent a significantly longer time in the area with the tube containing the ball of yarn than in the area with the empty tube (Fig. 2G, *F*_2,60_ = 3.944, p = 0.025; cage-mate in tube: *t*_9_ = −3.395, p = 0.007; ball of yarn in tube: *t*_9_ = −2.512, p = 0.029; opaque tube: *t*_9_ = −1.008, p = 0.339).

Next, we examined whether the subject mice would engage in rescue-like behaviour even when the inside of the tube could not be seen (Fig. 2B). We covered the tube containing a ball of yarn with white tape. We observed that the subject mice opened the paper lid of the opaque tube containing the ball of yarn (Fig. 2F, Supplementary Video S2). The latency to lid-opening was equivalent in the cases when the inside of the tube was not visible and when the cage-mate was in the tube (Fig. 2F). There were no significant differences between the time spent in the area with the opaque tube and the tube containing the cage-mate (Fig. 2G). These results indicate that the mice visually assess the condition of the tube.

### Rescue-like behaviour depends on the location of the entry tube

**Figure 3:**
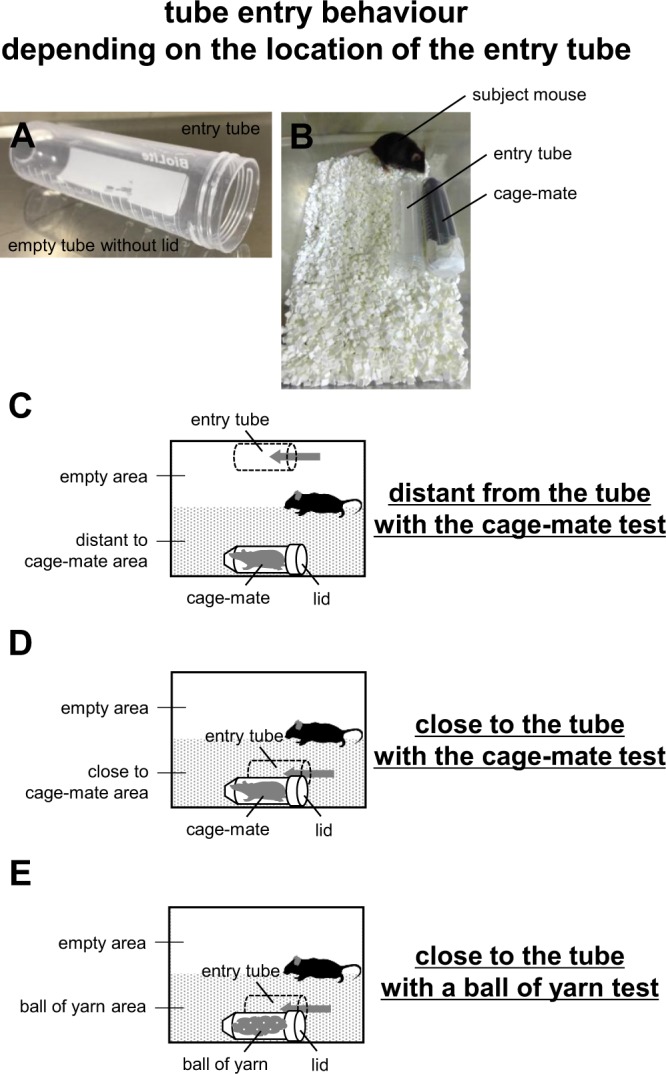
Schematic diagram of tube entry tests depending on the location of the tube. (**A**) A sample image of the entry tube. (**B**) A sample picture of the setup with the entry tube close to the tube containing the cage-mate in the new home cage. (**C**) Schematic diagram of the setup with the entry tube distant from the tube containing the cage-mate. (**D**) Schematic diagram of the setup with the entry tube close to the tube with the cage-mate. (**E**) Schematic diagram of the setup showing the entry tube close to the tube with a ball of yarn.

In the rescue-like behaviour test, the subject mouse often enters the tube after rescuing the cage-mate mouse. Therefore, we examined whether mice were interested in the tube itself. In this test, an empty tube without a lid was prepared, and the subject mouse was allowed to enter and exit freely (entry tube) (Fig. 3A).

**Figure 4:**
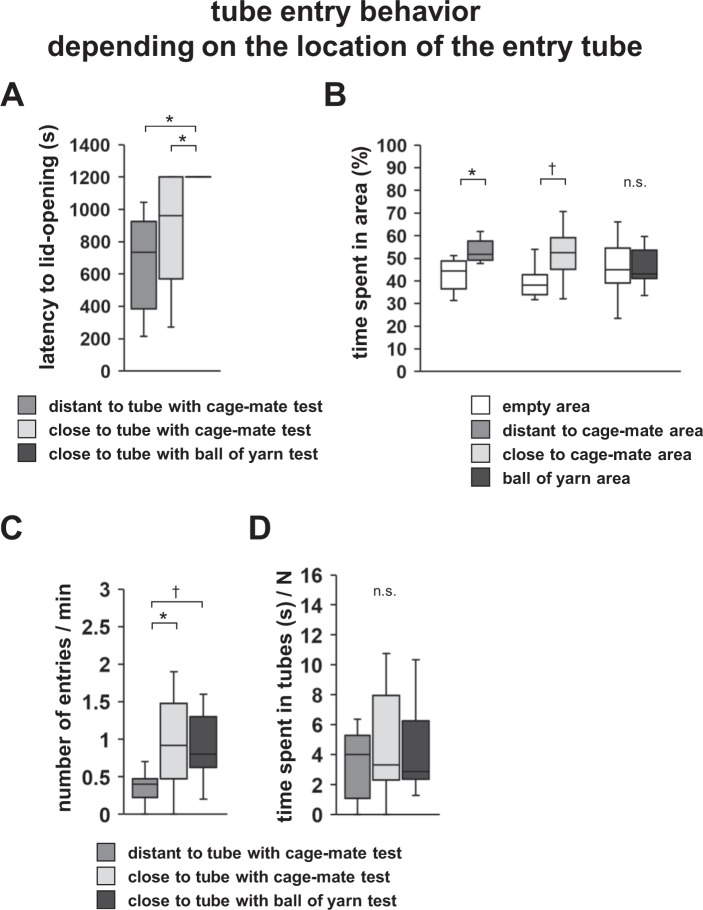
Tube entry tests depending on the location of the tube. (**A**) Comparison of the latency to lid-opening in the three tests. (**B**) Comparison of the time spent in each area in the three tests. (**C**) Comparison of the number of entries to the empty tube in the three tests. (**D**) Comparison of the time spent in the empty tubes in the three tests. All data are presented as box plots. Statistical significance is represented as ^†^p < 0.1 and *p < 0.05. n.s., not significant. The p values were calculated by one-way repeated measures ANOVA (**A**,**C**,**D**) and paired *t-*test (**B**). n = 10 animals per test.

First, we investigated the influence of the distance on rescue-like behaviour by placing this entry tube close to or distant from the constrained cage-mate (Fig. 3C,D). We observed that the subject mice opened the paper lids of tubes containing cage-mate mice in both conditions (Fig. 4A, Supplementary Video S3). No significant difference in the latency to lid-opening was detected between tubes placed close to and far from the constrained cage-mate (Fig. 4A, *F*_1,9_ = 167.481, p < 0.001; distant vs. close to cage-mate: p = 1.0; distant vs. close to ball of yarn: p = 0.039; close to cage-mate vs. close to ball of yarn: p = 0.045). In the distant-to-tube test, the subject mice spent a significantly longer time in the area with the tube containing the cage-mate than in the area with the entry tube (Fig. 4B, *F*_1,60_ = 2.385, p = 0.102; distant to the tube with the cage-mate: *t*_9_ = −2.87, p = 0.019; close to the tube with the cage-mate test: *t*_9_ = −1.924, p = 0.086; distant to the tube with a ball of yarn test: *t*_9_ = −0.079, p = 0.939). In the close-to-the-tube test, subject mice tended to spend a longer time in the area with the tube containing the cage-mate than in the empty area (Fig. 4B). We compared the number of entries to the entry tube when this entry tube was close to or distant from the constrained cage-mate. The number of entries was significantly higher for the entry tube close to the cage-mate than for the one distant from the cage-mate (Fig. 4C, *F*_1,9_ = 21.475, p = 0.001; distant vs. close to cage-mate: p = 0.017; distant vs. close to the ball of yarn: p = 0.092; close to the cage-mate vs. close to the ball of yarn: p = 0.631). There were no significant differences between the time spent in the entry tube between both conditions (Fig. 4D, *F*_1,9_ = 8.904, p = 0.015; distant vs. close to the cage-mate: p = 0.216; distant vs. close to the ball of yarn: p = 0.392; close to the cage-mate vs. close to the ball of yarn: p = 0.364). These results suggest that the interest in the entry tube increases when the cage-mate is nearby.

Next, we placed an entry tube close to a tube with a ball of yarn (Fig. 3E). We observed that subject mice did not open the paper lid of this tube (Fig. 4A). The subject mice spent almost the same amount of time in both areas (Fig. 4B). The number of entries tended to be higher for the entry tube close to the ball of yarn than for the one distant from the cage-mate (Fig. 4C). There were no significant differences between the time spent in the entry tube compared with other conditions (Fig. 4D). These results suggest the possibility that mice show interest in the entry tube even when a cage-mate is not present in a nearby tube.

### Rescue-like behaviour towards two cage-mates constrained inside tubes

**Figure 5:**
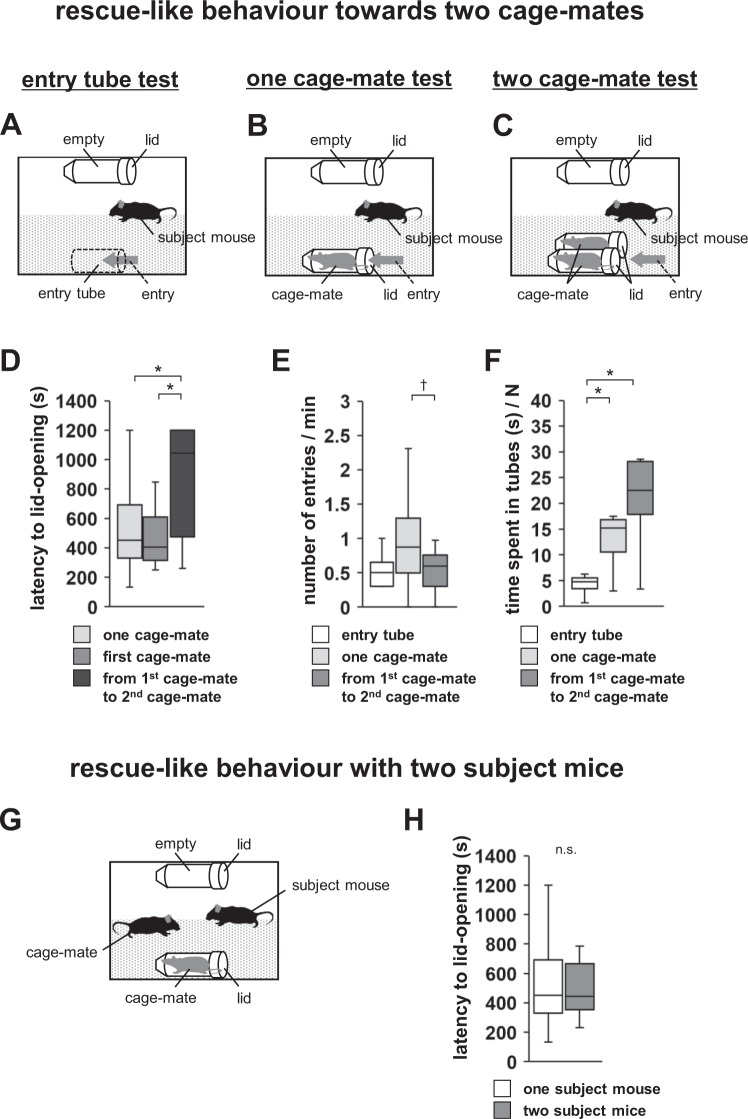
Rescue-like behaviour test towards two cage-mates constrained inside tubes. (**A**–**C**) Schematic diagrams of the tests. (**C**) Two tubes containing cage-mate mice on one side of the cage. Rescue-like behaviour test for two cage-mates: latency to lid-opening (**D**), number of entries to the tubes (**E**), and time spent in tubes (**F**) in this test. (**G**) Schematic diagram of the rescue-like behaviour test with two subject mice. (**H**) Comparison of the latency to lid-opening. Statistical significance is represented as: ^†^p < 0.1 and *p < 0.05. n.s., not significant. The p values were calculated by one-way repeated measures ANOVA (**D**–**F**,**H**). n = 10 animals per test.

In additional experiments, we prepared three transparent tubes, two containing cage-mate mice and the third containing nothing (Fig. 5C). We examined whether the subject mice showed rescue-like behaviour towards the two cage-mate mice simultaneously. We compared these results with those obtained using one cage-mate and an entry tube (Fig. 5A,B). No significant difference was detected between the latency to lid-opening with one cage-mate and two cage-mates (Fig. 5D, Supplementary Video S4, *F*_1,9_ = 48.016, p < 0.001; entry tube vs. one cage-mate: p = 0.289; entry tube vs. from 1^st^ cage-mate to 2^nd^ cage-mate: p = 0.024; one cage-mate vs. from 1^st^ cage-mate to 2^nd^ cage-mate: p = 0.003). The subject mouse took a long time to open the lid of the second tube (Fig. 5D). Subject mice showed no significant differences in the number of tube entries in these tests (Fig. 5E, *F*_1,9_ = 39.512, p < 0.001; entry tube vs. one cage-mate: p = 0.283; entry tube vs. from 1^st^ cage-mate to 2^nd^ cage-mate: p = 0.526; one cage-mate vs. from 1^st^ cage-mate to 2^nd^ cage-mate: p = 0.051). Subject mice spent more time in tubes recently opened by them than in open entry tubes (Fig. 5F, *F*_1,9_ = 23.740, p = 0.005; entry tube vs. one cage-mate: p = 0.028; entry tube vs. from 1^st^ cage-mate to 2^nd^ cage-mate: p = 0.015; one cage-mate vs. from 1^st^ cage-mate to 2^nd^ cage-mate: p = 0.229).

### Rescue-like behaviour of two subject mice at the same time

To find out why the latency to open the lid of the second tube increased, we placed two subject mice and examined the latency to open the lid of one cage-mate mouse (Fig. 5G). We observed that the subject mice opened the paper lid of the tube containing the cage-mate (Supplementary Video S5). No significant difference in the latency to lid-opening was detected between one subject mouse and two subject mice (Fig. 5H, *F*_1,9_ = 0.801, p = 0.394). These results indicate that mice show more interest in the tube itself due to the prior presence of the cage-mate in the tube.

## Discussion

In this study, we clarified that the crowding behaviour recognized as rescue-like behaviour in mice is not based on empathy but is instead related to the social contact with the cage-mate and an interest in the tube. Mice visually recognize the tube content and decide whether to open the lid or not. The results of this study indicate that mice want to be close to the cage-mates and may even prefer to show the same behaviours as the cage-mates. This study suggests that rodent lid-opening behaviour cannot prove the presence or absence of rodent prosocial behaviour.

We administered oxytocin to mice and assessed their tube lid-opening behaviour in comparison to mice treated with physiological saline. Oxytocin reportedly enhances empathy^28–31^, and many studies have reported that mice also have the ability to empathize^17,18,32^. Oxytocin-treated mice recognized the state of fear in their cage-mates, and due to their increased empathy, they exhibited an increased probability and time of immobility^27^. In a previous report, rescue-like behaviour of rats was reported to be based on empathy^8^. If rescue-like behaviour was based on empathy, administration of oxytocin could be expected to change it. However, in the present study, an oxytocin effect on rescue-like behaviour of mice was not confirmed. It is unclear whether oxytocin enhanced empathy or transmission of fear in the previously published rat study. However, our experimental results suggest that unlike these previously reported findings, rescue-like behaviour in mice may not be based on empathy.

Mice did not open the tube lid if they could visually confirm that the tube contained a ball of yarn and not the cage-mate. However, if a mouse could not visually confirm what was inside the tube, it opened the tube lid. These results indicate that mice visually assessed the tube content. Mice have been shown to be capable of seeing the behaviour of others^17,18^. Therefore, mice show an interest in cage-mates exhibiting abnormal behaviour^33^. Similarly, in a previous study on rescue-like behaviour in rats, it has been reported that rats do not show rescue-like behaviour on cylinders containing stuffed animals instead of cage-mates^5^. In the absence of a cage-mate in the cylinder, rats do not open the lid of the device, but this is believed to be due to the lack of screams from the restrained conspecifics^5,34^. In the current study, mice showed lid-opening behaviour despite the absence of conspecifics in opaque tubes. Although the rat behaviour of releasing other individuals from painful conditions despite the absence of incentives has been suggested to be prosocial behaviour^5,6,8^, it is clear that the lid-opening in itself cannot be interpreted as prosocial behaviour because mice in the present study opened also the lids of opaque tubes. This experimental result shows that the lid-opening behaviour, which is a rescue-like behaviour, is not driven by empathy but by an interest in tubes containing a mouse.

Subject rats were often found to enter the restraint device after showing rescue-like behaviour according to the movie materials of rescue-like behaviour in rats published by Ben-Ami *et al*. and Sato *et al*.^5,6^. Similarly, in the rescue-like behaviour shown in our previous report, subject mice entered the tube after opening the lid of the tube restraining the other individual^26^. These results indicate that mice and rats may be interested in restraints of conspecifics. In the present experiments, when there was an empty tube without a lid in the experimental environment, mice entered it several times. This mouse behaviour was augmented when the cage-mate was near the empty tube in comparison with the cases where the cage-mate was farther away. Mice are social animals and prefer to be with other mice^35–37^. It is also known that mice and rats have a strong tendency to follow conspecifics^37,38^. Rats show a tendency to select the same feeding site as that selected by conspecifics^39,40^. The present results show that when their cage-mate is restrained in a tube, mice are increasingly interested in the tube itself.

Interestingly, when a subject mouse encountered two restrained mice, the latency of opening the lid of the second tube was significantly longer than that for opening the lid of the first tube. In addition, some mice could not open the lid of the second tube within the test time. This increase in latency was not influenced by the presence of two subject mice that could freely move in the cage. The subject mouse still stayed in the tube that contained the first mouse for a long time. This could be attributed to the possibility that the interest in entering the tube that contained the first cage-mate was higher than that for opening the second lid. If the rescue-like behaviour of rodents was based on empathy as suggest in previous reports^5,6^, the latency to open the lid should not differ between the first and the second cage-mates. Previous reports in rats suggest that rat rescue-like behaviour is based on empathy, and rats are reported to give priority to rescue-like behaviour rather than obtaining chocolate as a reward^5,6^. However, in the current study, rather than rescuing the second cage-mate, the subject mouse entered the first tube and prioritized spending time in it. Unlike previous reports, the results obtained with mice in this study indicate that the motivation for rescue-like behaviour is an interest in tubes and the tendency to follow conspecifics. Indeed, in rat rescue-behaviour tests, the rats showed greater interest in cylinders containing conspecifics^23^. The presence of conspecifics is generally known to attract interest in animals. These behaviours are called “stimulus and local enhancement”^41,42^. The learning effect of the subject rat improves on observing the behaviour of its conspecifics^43^. As a result of the social approach or the stimulus and local enhancement in response to a restrained mouse, it is considered that subject mice open the tube lid, enter the tube, and stay there in order to experience the condition of their conspecifics. In fact, in an environment without cage-mates, subject mice stayed in the empty tube for only a short time. This result of the current study suggests that the empty tube entry behaviour in mice is in agreement with the stimulus and local enhancement theory and represents a follow-up on conspecifics. That is, it is suggested that the rescue-like behaviour in mice is not based on empathy but is instead based on the social interest and the tendency of mice to follow-up on conspecifics.

Ben-Ami *et al*. suggested empathic hypotheses, whereas Silberberg *et al*. suggested social-contact hypotheses^22–24^. Our results support the social-contact hypothesis of Silberberg *et al*. In addition to this hypothesis, the present study shows that the rescue-like behaviour of mice is based on social contacts and interests in the tube. This research fully elucidates the motives underlying the rescue-like behaviour shown by mice and rats. Further experiments will be needed to confirm these hypotheses and to determine the motive for rescue-like behaviour in rodents. In addition, this study does not deny that mice exhibit prosocial behaviour. However, the study findings indicate the need for a new experimental method that does not assess lid-opening behaviour for constrained conspecifics in order to investigate the presence or absence of prosocial behaviour in rats and mice.

## Conclusion

We suggest that the murine behaviour of helping conspecifics constrained to the tube was not based on empathy but was instead driven by the need for social contact and an interest in restraint devices. The results of this research are useful for establishing a new experimental method to investigate the presence or absence of prosocial behaviour of rodents.

## Methods

### Animals

All animal experiments were performed in accordance with the U.S. National Institutes of Health (NIH) Guide for the Care and Use of Laboratory Animals (NIH Publication No. 80-23, revised in 1996) and approved by the Committee for Animal Experiments at Kawasaki Medical School Advanced Research Center. All efforts were made to minimise the number of animals used and their suffering. Animals were purchased from Charles River Laboratories (Kanagawa, Japan) and housed in cages (five animals per cage) with food and water provided *ad libitum* under a 12-h light/dark cycle at 23 °C–26 °C. We used C57BL/6N male mice aged 10 weeks. All behavioural tests were conducted in behavioural testing rooms between 08.00 and 18.00 h during the light phase of the circadian cycle. After the tests, all equipment was cleaned with 70% ethanol and super hypochlorous water to prevent bias based on olfactory cues. Behavioural tests were performed according to the test order described below.

### Training in opening the paper lid

Mice were subjected to training in opening the paper lid by being constrained inside transparent plastic cylinders (50-mL tubes, diameter 3 cm). The front of the tube was closed with a paper lid, whereas its rear was closed with a plastic lid. The tube was placed inside a new home cage, and after the mice managed to break through the paper lid and exit the tube, they were allowed to act freely for 5 min. We practised this exercise three times a day for 2 days. All mice opened the paper lid and exited the tubes.

### Rescue-like behaviour test

We prepared 50-mL tubes with a cut tip. We closed the back of the empty tube and of the tubes containing constrained mice with a paper lid (Fig. 1A,B). In this test, a familiar mouse (cage-mate) was placed into one of the transparent tubes located at the sides of a new home cage. We placed an empty tube on the other side (Fig. 1C,D). The constrained cage-mate mice were also trained to open the paper lid. The subject mouse was placed at the centre and allowed to explore the entire home cage for a 90-min session. The latency to open the lid of the tube containing the cage-mate mouse was measured. After opening the lid, mice were allowed to act freely for 5 min. The mice had simultaneously the opportunity to open the paper lid of the empty tube. We performed the test once a day for 10 days. We used mice that had learned to open the lid of the tube containing the cage-mate mouse in all experiments. Mice that did not learn how to open the lid were excluded from the analysis. Each mouse was tested once per test. n = 10 animals per test. The data were recorded on video.

### Oxytocin administration

A computer randomly assigned trained mice to two groups. Oxytocin (100 μg/kg, dissolved in sterile saline; 335-40841; FUJIFILM Wako Pure Chemical Corporation, Tokyo, Japan) or vehicle (1 mL/kg; saline) solutions were intraperitoneally injected into the mice. These mice underwent a rescue-like behaviour test 30 min after injection.

### Testing rescue-like behaviour depending on the tube condition

In this test, a cage-mate mouse or a ball of yarn resembling a mouse (Fig. 2A) were placed into one of the transparent tubes located at the side of a new home cage. In additional experiments, an opaque tube was prepared by covering a 50-mL tube with white tape (Fig. 2B), and this tube was placed on the same side of a new home cage. We placed an empty tube on the other side of this cage. The subject mouse was placed at the centre and allowed to explore the entire home cage for a 20-min session. The test was terminated when the mouse opened the paper lid. The term “empty area” refers to the half of the home cage containing the empty tube (Fig. 2C–E). The latency to lid-opening and the amount of time spent in each area during the 20-min sessions were measured. The time spent in each area was measured only before one of the tubes was opened. Data were recorded on video and analysed using video tracking software (ANY-MAZE, Stoelting Co., Wood Dale, IL).

### Testing tube entry behaviour depending on the location of the entry tube

We prepared an empty 50-mL entry tube without a paper lid (Fig. 3A). We also prepared transparent tubes containing cage-mate mice or a ball of yarn and closed one side with a paper lid. These tubes were located at opposite sides of a new home cage (Fig. 3B–E). The entry tube was placed close to or distant from the other tube (Fig. 3B–E). The latency to lid-opening and the amount of time spent in each area during the 20-min sessions were measured. The time spent in each area was measured only before the tube was opened. We also analysed the number of entries into the entry tube and the time (in s) spent in the entry tube. The data were recorded on video and analysed using the ANY-MAZE software.

### Testing rescue-like behaviour towards two cage-mates constrained inside tubes

We prepared an empty 50-mL entry tube without a paper lid (Fig. 3A). We also prepared transparent tubes containing cage-mate mice and closed one side with a paper lid. In test 1, we placed the entry tube on one side and an empty tube on the opposite side of the cage (Fig. 5A). In test 2, we placed the transparent tube containing the cage-mate mouse on one side and an empty tube on the other (Fig. 5B). In test 3, we placed two transparent tubes containing cage-mate mice on one side and an empty tube on the other (Fig. 5C). The latency to lid-opening (in s), the number of tube entries, and the time spent in each tube (in s) during the 20-min sessions were measured. The data were recorded on video and analysed using the ANY-MAZE software.

### Testing rescue-like behaviour of two subject mice at the same time

A cage-mate mouse was placed into one of the transparent tubes located at the sides of a new home cage. We placed an empty tube on the other side (Fig. 5G). Two subject mice were placed at the centre and allowed to explore the entire home cage for a 20-min session. The latency to lid-opening of the tube containing the cage-mate mouse was measured.

### Statistical analysis of behavioural tests

Statistical analyses were conducted using SPSS software (IBM Corp, Tokyo, Japan). If the variables were found to be non-normally distributed, a non-parametric analysis was used. We used the paired *t*-test, one-way repeated ANOVA, or two-way repeated measures ANOVA. A p-value < 0.05 was regarded as statistically significant. Data are shown as box plots.

## Supplementary information

Supplementary Video S1

Supplementary Video S2

Supplementary Video S3

Supplementary Video S4

Supplementary Video S5

Supplementary Figure Legends

## Data Availability

All relevant data are within the manuscript.
