# Supplementary material for: Rescue-like Behaviour in Mice is Mediated by Their Interest in the Restraint Tool
Source: Sci Rep. 2019 Aug 2;9:10648. doi: 10.1038/s41598-019-46128-5 (PMC6677799; doi:10.1038/s41598-019-46128-5)
Supplement: Supplementary file 6 — Supplementary Figure Legends [file 41598_2019_46128_MOESM6_ESM.docx]

1. ORIGINAL RESEARCH ARTICLE

**Rescue-like Behaviour in Mice is Mediated by Their Interest in the Restraint Tool**

**Hiroshi Ueno^1,^*, Shunsuke Suemitsu^2^, Shinji Murakami^2^, Naoya Kitamura^2^,　Kenta Wani^2^, Yu Takahashi^2^, Yosuke Matsumoto^3^, Motoi Okamoto^4^, Takeshi Ishihara^2^**

1. 1 Department of Medical Technology, Kawasaki University of Medical Welfare, Okayama, 701-0193, Japan
2. 2 Department of Psychiatry, Kawasaki Medical School, Kurashiki, 701-0192, Japan
3. 3 Department of Neuropsychiatry, Graduate School of Medicine, Dentistry and Pharmaceutical Sciences, Okayama University, Okayama, 700-8558, Japan
4. 4 Department of Medical Technology, Graduate School of Health Sciences, Okayama University, Okayama, 700-8558, Japan

**Author information**

Hiroshi Ueno, PhD. E-mail: dhe422007@s.okayama-u.ac.jp

Shunsuke Suemitsu, PhD, MD. E-mail: ssue@med.kawasaki-m.ac.jp

Shinji Murakami, PhD, MD. E-mail: muraka@med.kawasaki-m.ac.jp

Naoya Kitamura, PhD, MD. E-mail: n-kitamura@med.kawasaki-m.ac.jp

Kenta Wani, PhD, MD. E-mail: k-wani@med.kawasaki-m.ac.jp

Yu Takahashi, MD. E-mail: yuuu.takahashi@gmail.com

Yosuke Matsumoto, PhD, MD. E-mail: ymatsumoto@okayama-u.ac.jp

Motoi Okamoto, PhD, MD. E-mail: mokamoto@md.okayama-u.ac.jp

Takeshi Ishihara, PhD, MD. E-mail: t-ishihara@med.kawasaki-m.ac.jp

*Corresponding author.

1. Hiroshi Ueno, PhD.
2. Address: Department of Medical Technology, Kawasaki University of Medical Welfare, 288, Matsushima, Kurashiki, Okayama, 701-0193, Japan
3. Phone: +81-86-462-1111, Fax: +81-86-462-1193
4. E-mail address: dhe422007@s.okayama-u.ac.jp (H. Ueno)

**Supplementary Figure Legends**

**Supplementary Video S1** Rescue-like behaviour test for a ball of yarn.

This is a 16× speed movie.

**Supplementary Video S2** Rescue-like behaviour test for the opaque tube.

This is a 4× speed movie, and the first 900 s of the test has been deleted.

**Supplementary Video S3** Rescue-like behaviour in a setup with the entry tube close to the tube with the cage-mate.

This is a 16× speed movie.

**Supplementary Video S4** Rescue-like behaviour test towards two cage-mates constrained inside tubes.

This is a 16 × speed movie.

**Supplementary Video S5** Rescue-like behaviour test with two subject mice.

This is an 8× speed movie.
